# Association between obesity phenotypes and non-alcoholic fatty liver: a large population- based study

**DOI:** 10.1186/s12902-024-01630-4

**Published:** 2024-06-25

**Authors:** Farid Najafi, Yahya Pasdar, Mehdi Moradi Nazar, Mitra Darbandi

**Affiliations:** 1 Research Center for Environmental Determinants of Health (RCEDH), Health Institute, Kermanshah University of Medical Sciences, Kermanshah, Iran; 2 Student Research Committee, Kermanshah University of Medical Sciences, Kermanshah, Iran

**Keywords:** Fatty liver, Obesity, Metabolic syndrome, PERSIAN

## Abstract

**Background:**

The aim of this study was to examine the association between different metabolic obesity phenotypes and the non-alcoholic fatty liver disease (NAFLD).

**Methods:**

This cross-sectional analysis utilized data from the baseline phase of the Ravansar non-communicable diseases (RaNCD) cohort study, which involved 8,360 adults. Participants with a Fatty Liver Index (FLI) score of ≥ 60 was classified as having NAFLD. The FLI score was calculated using liver non-invasive markers and anthropometric measurements. Participants were categorized into four phenotypes based on the presence or absence of metabolic syndrome and obesity. Logistic regression analysis was used to evaluate the association of NAFLD and obesity phenotypes.

**Results:**

According to the FLI index, the prevalence of NAFLD was 39.56%. Participants with FLI scores of ≥ 60 had higher energy intake compared to those in the FLI < 60 group (*P* = 0.033). In subjects with metabolically unhealthy phenotypes, the level of physical activity was lower compared to those with metabolically healthy phenotypes. The risk of NAFLD in males with the metabolically healthy-obese phenotype increased by 8.92 times (95% CI: 2.20, 15.30), those with the metabolically unhealthy-non-obese phenotype increased by 7.23 times (95% CI: 5.82, 8.99), and those with the metabolically unhealthy-obese phenotype increased by 32.97 times (95% CI: 15.70, 69.22) compared to the metabolically healthy-non-obese phenotype. Similarly, these results were observed in females.

**Conclusion:**

This study demonstrated that the risk of NAFLD is higher in individuals with metabolically healthy/obese, metabolically unhealthy/non-obese, and metabolically unhealthy/obese phenotypes compared to those with non-obese/metabolically healthy phenotypes.

## Introduction

Non-alcoholic fatty liver disease (NAFLD) refers to the accumulation of fat in the liver without excessive alcohol intake. NAFLD is closely tied to conditions like obesity, type 2 diabetes, and dyslipidemia. NAFLD is the most common chronic liver disease, and its prevalence is rising in parallel with the increasing rates of obesity and diabetes. NAFLD can progress from a simple fat buildup to more advanced forms like non-alcoholic steatohepatitis (NASH), which can lead to cirrhosis, liver failure, and liver cancer [1–3]. NAFLD is also associated with an elevated risk of cardiovascular disease (CVD), chronic kidney disease (CKD), and other metabolic disorders, posing a significant financial burden on healthcare systems [4, 5]. The NAFLD is a prevalent metabolic disease worldwide, with an estimated prevalence of 25% globally and 27% in Asia [6, 7]. Early diagnosis, prevention, and control of NAFLD are crucial. Non-invasive indices such as fatty liver index (FLI), hepatic steatosis index (HSI), non-alcoholic steatohepatitis (NASH) Score, and Steato Test (ST) are used to predict fatty liver. FLI, which is a composite equation of waist circumference (WC), body mass index (BMI), gamma glutamyl transpeptidase (GGT), and triglycerides, is particularly useful in population-based studies [8, 9]. A study conducted in Italy found that the FLI had a predictive power of 82% (Area under the Curve (AUC) :0.82). The study also revealed that the cut-off point for the FLI is smaller in women compared to men [10].

Studies have shown an association between MetS and fatty liver, with a higher prevalence of fatty liver observed in patients with MetS [9, 11, 12]. Obesity is also closely related to NAFLD, with approximately 80% of obese individuals affected by NAFLD [13, 14]. An increase in visceral fat and inflammation plays a significant role in NAFLD development, with those with the highest amount of visceral fat and the largest adipocyte diameter at the highest risk of developing NAFLD among obese individuals [15–20].

To evaluate the simultaneous effect of obesity and metabolic disorders on fatty liver, individuals are classified into four phenotypes based on the presence or absence of MetS and obesity. The obesity phenotype is divided into four groups based on the presence or absence of BMI and MetS [21]. A good correlation between FLI as a marker of NAFLD and abnormal metabolic phenotypes has been shown in all BMI ranges [22]. However, research on the association between fatty liver and metabolic phenotypes is limited. Therefore, this study aims to assess the association between metabolic obesity phenotypes and NAFLD.

## Methods

### Study population

**Fig. 1:**
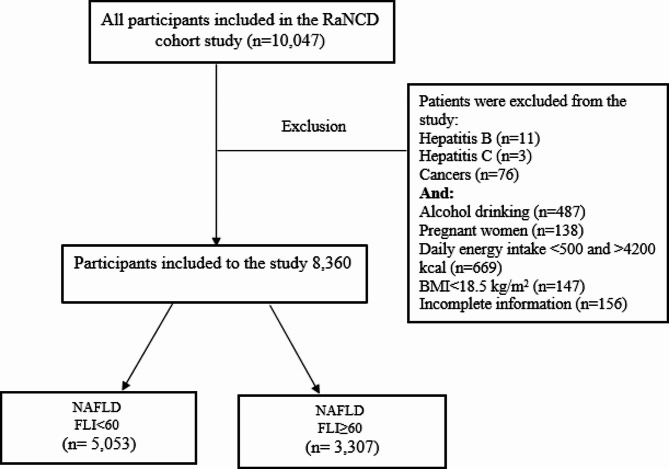
Flow chart of study

The study population consisted of participants from the baseline phase of the Ravansar non-communicable diseases (RaNCD) cohort study, which is a part of the PERSIAN (Prospective Epidemiological Research Studies in Iran) researches [23]. The RaNCD study is a 15-year prospective epidemiological study that started in 2014 [24]. The sample size for the baseline phase was 10,047 participants, aged between 35 and 65 years. Certain exclusion criteria were applied, resulting in a final sample size of 8360 participant’s patients with hepatitis B (*n* = 11), hepatitis C (*n* = 3), cancers (*n* = 76), alcohol drinking (*n* = 487), pregnancy (*n* = 138), energy intake < 500 and > 4200 kcal/day (*n* = 669), BMI < 18.5 kg/m^2^ (*n* = 147) and incomplete information (*n* = 156) were excluded from the study. Thus, 8360 participants were included in the present study **(**Fig. 1**).**

### Measurements

The RaNCD study protocol has been previously published [24]. Further information is available at http://persiancohort.com.Various measurements were taken during the study. Questionnaire information, including demographics, lifestyle, and physical activity, was collected through face-to-face interviews using digital questionnaires. Current smoking status was defined as consuming at least 100 cigarettes per year. Socio-economic status (SES) was determined using principal component analysis (PCA) based on education level, amenities, and place of residence [25]. The level of physical activity was evaluated using the PERSIAN cohort questionnaire and divided into three groups based on the amount of metabolic equivalent of task (MET) per hour per day (low: 24-36.5, medium: 36.6–44.4, and high: ≥ 44.5) [26]. Body composition and anthropometric indices such as BMI, body fat mass (BFM), visceral fat area (VFA), waist circumference (WC) and waist-to-hip ratio (WHR) were measured using a Bio-Impedance BIA analyzer. The liver enzymes including alanine aminotransferase (ALT), aspartate transaminase (AST) and GGT were measured after 12 h of fasting.

### Fatty liver index

The fatty liver index (FLI) was calculated based on four variables: triglycerides (TG), BMI, GGT and WC. FLI ranges from 0 to 100, with scores below 60 indicating the absence of fatty liver and scores equal to or above 60 indicating the presence of fatty liver [8]. FLI was first defined in 2006 by Bedogni et al. based on four variables as predictors for fatty liver according to the following Eq. [8]:

$${\text{FLI}} = \frac{{\left( {{{\text{e}}^{\scriptstyle 0.953 \times {\text{log}}\left( {\text{e}} \right)\left( {{\text{TG}}} \right) + 0.139 \times {\text{BMI}} + 0.718 \hfill \atop \scriptstyle \times {\text{log}}\left( {\text{e}} \right)\left( {{\text{GGT}}} \right) + 0.053 \times {\text{WC - }}15.745 \hfill} }} \right)}}{{\left( {1 + {{\text{e}}^{\scriptstyle 0.953 \times {\text{log}}\left( {\text{e}} \right)\left( {{\text{TG}}} \right) + 0.139 \times {\text{BMI}} + 0.718 \hfill \atop \scriptstyle \times {\text{log}}\left( {\text{e}} \right)\left( {{\text{GGT}}} \right) + 0.053 \times {\text{WC - }}15.745 \hfill} }} \right)}} \times 100$$

### Obesity phenotypes

General obesity is defined based on BMI. The people with BMI above 30 kg/m2 as obese. International Diabetes Federation (IDF) criteria for metabolic status are considered. Participants were classified into four obesity phenotypes based on their BMI and metabolic profile: metabolically healthy/non-obese, metabolically unhealthy/non-obese, metabolically healthy/obese, and metabolically unhealthy/obese.

### Statistical analysis

Statistical analysis was performed using Stata software version 14.2 (Stata Corp, College Station, TX, USA). Descriptive statistics were used to summarize continuous variables (mean and standard deviation) and qualitative variables (frequency and percentage). T-tests and chi-square tests were used to compare differences between groups (FLI < 60 and FLI ≥ 60). One-way ANOVA and chi-square tests were used to compare differences between the four obesity phenotypes. Logistic regression analysis was conducted to determine the association between obesity phenotype and NAFLD. Multivariate logistic regression analysis was performed to assess the relationship between demographic, lifestyle, anthropometric characteristics, comorbidities, and NAFLD. Crude and adjusted logistic regression models were used to evaluate the association between metabolic obesity phenotype and NAFLD, with adjustments made for significant variables identified in the multivariate model. All estimates were reported with a 95% confidence interval, and a significance level of *P* < 0.05 was considered statistically significant.

## Results

### Basic characteristics of study participants according to fatty liver Inex

A total of 8,360 participants (3,569 men and 4,791 women) with an average age of 47.60 ± 8.28 years were included in this study. The prevalence of NAFLD, as determined by the FLI, was found to be 39.56%. The prevalence of NAFLD was significantly higher in women compared to men (40.99% vs. 37.63%, *P* = 0.002). Participants with FLI ≥ 60 had lower levels of physical activity (*P* < 0.001) and higher energy intake (2529.82 ± 740.28 vs. 2494.52 ± 743.30 kcal/day, *P* = 0.033) compared to those with FLI < 60 **(**Table 1**)**.

**Table 1:** Baseline characteristics of the study participants according to fatty liver index (*n* = 8,360)

Characteristic	Total	FLI < 60	FLI ≥ 60	*P* value *
	Mean ± SD / *n* (%)			
N (%)	8,360	5,053 (60.44)	3,307 (39.56)	-
Age (year)	47.60 ± 8.28	47.12 ± 8.41	48.34 ± 8.02	< 0.001
**Sex**				
Men	3569 (42.69)	2226 (44.05)	1343 (40.61)	0.002
Women	4791 (57.31)	2827 (55.95)	1964 (59.36)	
**Residency**				
Urban	4954 (59.26)	2896 (57.31)	2058 (62.23)	0.001
Rural	3406 (40.74)	2157 (42.69)	1249 (37.77)	
**Socioeconomic status**				
Low	2871 (34.34)	1806 (35.74)	1065 (32.20)	0.004
Moderate	2796 (33.44)	1658 (32.81)	1138 (34.41)	
High	2693 (32.21)	1589 (31.45)	1104 (33.38)	
**Physical activity**				
Low	2546 (30.45)	1367 (27.05)	1179 (35.65)	< 0.001
Moderate	4118 (49.26)	2487 (49.22)	1631 (49.32)	
High	1696 (20.29)	1199 (23.73)	497 (15.03)	
**Smoking status**				
Current smoker	748 (8.95)	508 (10.05)	240 (7.26)	< 0.001
Former	645 (7.72)	350 (6.93)	295 (8.92)	
Never	6967 (83.34)	4195 (83.02)	2772 (83.82)	
**Comorbidity**				
Dyslipidemia	3654 (43.71)	1676 (33.17)	1978 (59.51)	< 0.001
T2DM	740 (8.85)	274 (5.42)	466 (14.09)	< 0.001
CVD	1484 (17.75)	704 (13.93)	780 (23.59)	< 0.001
Hypertension	1357 (16.23)	663 (13.12)	694 (20.99)	< 0.001
Renal failure	85 (1.02)	40 (0.79)	45 (1.36)	0.011
**Anthropometric indices**				
BMI (kg/m^2^)	27.69 ± 4.48	25.34 ± 3.04	31.28 ± 3.91	< 0.001
WHR	0.94 ± 0.06	0.92 ± 0.05	0.98 ± 0.05	< 0.001
BFM (kg)	25.62 ± 9.33	21.06 ± 6.61	32.60 ± 8.53	< 0.001
VFA (cm^2^)	125.72 ± 50.58	102.56 ± 39.48	161.11 ± 44.88	< 0.001
**Liver enzymes**				
AST (mg/dl)	21.22 ± 8.79	20.50 ± 7.61	22.33 ± 10.25	< 0.001
ALT (mg/dl)	24.45 ± 14.27	21.78 ± 11.04	28.55 ± 17.34	< 0.001
GGT (mg/dl)	24.36 ± 19.74	19.10 ± 10.50	32.47 ± 26.60	< 0.001
**Dietary intake**				
Energy intake (Kcal/day)	2508.48 ± 742.26	2494.52 ± 743.30	2529.82 ± 740.28	0.033
Whole grains (gr/day)	9.98 ± 0.85	9.57 ± 1.73	10.61 ± 0.22	< 0.001
Refined grains (gr/day)	502.17 ± 2.50	505.15 ± 2.10	497.61 ± 2.57	0.022
Dairy (gr/day)	442.05 ± 5.05	432.11 ± 4.87	457.24 ± 6.02	0.001
Vegetables (gr/day)	469.48 ± 3.5	455.12 ± 3.31	491.42 ± 4.10	0.001
Fruits (gr/day)	270.78 ± 2.84	261.13 ± 2.65	285.53 ± 3.28	< 0.001
Meat (gr/day)	72.96 ± 0.70	71.60 ± 0.66	75.04 ± 0.82	0.001
Egg (gr/day)	19.62 ± 0.27	20.43 ± 0.25	18.40 ± 0.31	< 0.001
Legumes (gr/day)	32.62 ± 0.30	32.88 ± 0.39	32.22 ± 0.48	0.288
Sweets and desserts (gr/day)	56.20 ± 0.52	58.03 ± 0.49	53.41 ± 0.61	< 0.001

*P-values were obtained t-test and Chi square

*Abbreviation* *CVD* cardiovascular disease; *T2DM* type 2 diabetes mellitus; *ALT* alanine aminotransferase; *AST* aspartate aminotransferase; *GGT* gamma-glutamyl transferase; *BMI* body mass index; *WHR* waist hip ratio; *VFA* visceral fat area; *BFM* Body fat mass; *SES* socioeconomic status

### Baseline characteristics of the study participants according to phenotype obesity

In individuals with metabolically unhealthy phenotypes, the level of physical activity was lower compared to those with metabolically healthy phenotypes. Anthropometric indices and liver enzymes were significantly higher in individuals with metabolically unhealthy and obese phenotypes compared to the other three groups. The intake of daily energy, hydrogenated fats, total saturated fatty acids, cholesterol, and butter was lower among the obese/metabolically unhealthy group compared to the non-obese/metabolically healthy group **(**Table 2**).**

**Table 2:** Baseline characteristics of the study participants according to phenotype obesity (*n* = 8,360)

Characteristic	Metabolically healthy		Metabolically unhealthy				*P* value *
	Non-obese (*n* = 3,949)	Obese (*n* = 1,158)	Non-obese (*n* = 2,109)		Obese (*n* = 1,144)		
	Mean ± SD / *n* (%)						
Age (year)	46.36 ± 8.23	45.98 ± 7.51		49.98 ± 8.40		49.12 ± 7.77	< 0.001
**Sex**							
Men	1959 (49.61)	253 (21.85)		1045 (49.55)		312 (27.27)	< 0.001
Women	1990 (50.39)	905 (78.15)		1064 (50.45)		832 (72.73)	
**Socioeconomic status**							
Low	1373 (34.77)	374 (32.30)		717 (34.00)		407 (35.58)	0.008
Moderate	1287 (32.59)	404 (34.89)		684 (32.43)		421 (36.80)	
High	1289 (32.64)	380 (32.82)		708 (33.57)		316 (27.62)	
**Physical activity**							
Low	1082 (27.40)	355 (30.66)		700 (33.19)		409 (35.75)	< 0.001
Moderate	1858 (47.05)	642 (55.44)		1010 (47.89)		608 (53.15)	
High	1009 (25.55)	161 (13.90)		399 (18.92)		127 (11.10)	
**Smoking status**							
Never	3228 (82.14)	1044 (90.63)		1667 (79.42)		988 (86.74)	< 0.001
Current smoker	412 (10.48)	46 (3.99)		232 (11.05)		58 (5.09)	
Former smoker	290 (7.38)	62 (5.38)		200 (9.53)		93 (8.17)	
**Comorbidity**							
Dyslipidemia	980 (24.82)	278 (24.01)		1615 (76.58)		781 (68.27)	< 0.001
T2DM	66 (8.92)	31 (4.19)		387 (52.30)		256 (34.59)	< 0.001
CVD	258 (17.39)	121 (8.15)		671 (45.22)		434 (29.25)	< 0.001
Hypertension	224 (16.51)	68 (5.01)		647 (47.68)		418 (30.80)	< 0.001
Renal failure	24 (0.61)	7 (0.60)		34 (1.61)		20 (1.75)	< 0.001
**Anthropometric indices**							
BMI (kg/m^2^)	25.10 ± 2.87	33.12 ± 3.10		26.48 ± 2.33		33.36 ± 3.29	< 0.001
WHR	0.92 ± 0.05	0.99 ± 0.05		0.94 ± 0.05		0.99 ± 0.06	< 0.001
BFM (kg)	20.55 ± 6.32	36.69 ± 6.77		23.05 ± 5.27		36.70 ± 7.31	< 0.001
VFA (cm^2^)	99.11 ± 37.69	183.61 ± 33.34		112.91 ± 33.490		182.61 ± 35.03	< 0.001
Liver enzymes							
AST (mg/dl)	21.14 ± 8.34	20.42 ± 10.82		21.81 ± 8.47		21.21 ± 8.48	< 0.001
ALT (mg/dl)	22.81 ± 12.93	24.01 ± 15.53		26.73 ± 15.46		26.40 ± 14.24	0.001
GGT (mg/dl)	21.49 ± 17.10	23.33 ± 18.77		28.19 ± 23.04		28.27 ± 20.78	< 0.001
**Dietary intake**							
Energy intake (Kcal/day)	2538.45 ± 738.18	2515.82 ± 735.36		2469.36 ± 757.90		2469.72 ± 729.92	0.004
Whole grains (gr/day)	9.43 ± 0.20	10.25 ± 0.36		10.37 ± 0.27		10.93 ± 0.36	0.006
Refined grains (gr/day)	511.44 ± 2.34	480.36 ± 4.33		502.13 ± 3.21		492.29 ± 4.36	< 0.001
Dairy (gr/day)	418.38 ± 5.50	476.34 ± 10.15		451.66 ± 7.53		471.31 ± 10.22	< 0.001
Vegetables (gr/day)	442.84 ± 3.73	499.20 ± 6.89		480.01 ± 5.11		511.91 ± 6.94	< 0.001
Fruits (gr/day)	251.21 ± 2.99	306.96 ± 5.52		269.01 ± 4.10		304.99 ± 5.55	< 0.001
Meat (gr/day)	71.71 ± 0.75	70.04 ± 1.38		76.31 ± 1.02		74.05 ± 1.39	0.004
Egg (gr/day)	21.21 ± 0.28	17.84 ± 0.52		18.98 ± 0.39		17.13 ± 0.53	< 0.001
Legumes (gr/day)	32.61 ± 0.44	31.63 ± 0.81		33.51 ± 0.60		31.98 ± 0.82	0.236
Sweets and desserts (gr/day)	59.38 ± 0.55	56.73 ± 1.02		53.18 ± 0.76		50.26 ± 1.03	< 0.001
Soft drinks (gr/day)	35.34 ± 0.82	34.59 ± 1.52		34.86 ± 1.13		33.54 ± 1.52	0.774
Hydrogenated fats (gr/day)	19.90 ± 0.29	20.44 ± 0.54		18.37 ± 0.40		18.39 ± 0.54	0.008
Fatty acid total saturated (gr/day)	28.10 ± 0.15	29.03 ± 0.27		27.61 ± 0.20		27.62 ± 0.27	0.001
Fatty acid total trans (gr/day)	0.27 ± 0.01	0.28 ± 0.01		0.26 ± 0.02		0.25 ± 0.01	0.201
Cholesterol (gr/day)	271.63 ± 1.65	264.22 ± 3.05		269.62 ± 2.26		260.42 ± 3.10	0.005
Butter (gr/day)	3.75 ± 0.07	3.64 ± 0.13		3.27 ± 0.10		3.05 ± 0.13	0.001
Salt (gr/day)	4.26 ± 0.04	4.44 ± 0.08		4.06 ± 0.06		4.12 ± 0.08	0.007
Sugar (gr/day)	136.65 ± 0.60	144.21 ± 1.10		136.42 ± 0.82		140.28 ± 1.10	0.001

*P-values were obtained one-way ANOVA and Chi square.

*Abbreviation* *CVD* cardiovascular disease; *T2DM* type 2 diabetes mellitus; *ALT* alanine aminotransferase; *AST* aspartate aminotransferase; *GGT* gamma-glutamyl transferase; *BMI* body mass index; *WHR* waist hip ratio; *VFA* visceral fat area; *BFM* Body fat mass; *SES* socioeconomic status

### Association between the sociodemographic characteristics and fatty liver index

Higher SES was associated with 18% lower odds of NAFLD compared to lower SES. Moderate and high levels of physical activity were associated with 16% and 31% lower odds of NAFLD, respectively, compared to low physical activity. Current smokers had 27% lower odds of NAFLD compared to non-smokers. Increased BMI and visceral fat area (VFA) were associated with an increased risk of NAFLD (*P* < 0.001). Dyslipidemia, type 2 diabetes mellitus (T2DM), and MetS were significantly more prevalent in individuals with NAFLD. Using multiple analysis, the odds of NAFLD in women were significantly 83% lower than in men (OR: 0.17; 95%CI: 0.13, 0.22) **(**Table 3**).**

**Table 3:** Association between the baseline characteristics and fatty liver index by univariable and multiple logistic regression model

	OR (95% CI)		*P* value *
	Crude model	Adjusted model*	
Age (year)	1.02 (1.01,1.02)	1.02 (1.01, 1.03)	< 0.001
**Sex**			
Men	1	1	
Women	1.15 (1.05, 1.25)	0.17 (0.13, 0.22)	< 0.001
Socioeconomic status			
Low	1	1	
Moderate	1.16 (1.04, 1.29)	0.89 (0.74, 1.06)	0.206
High	1.17 (1.05, 1.31)	0.82 (0.68, 1.03)	0.049
**Physical activity**			
Low	1	1	
Moderate	0.76 (0.68, 0.84)	0.84 (0.70, 0.97)	0.042
High	0.48 (0.42, 0.547)	0.69 (0.55, 0.85)	0.001
**Smoking status**			
Never	1	1	
Former	1.27 (1.08, 1.50)	1.18 (0.90, 1.54)	0.228
Current smoker	0.71 (0.61, 0.840)	0.73 (0.56, 0.96)	0.033
**Comorbidity**			
Dyslipidemia	2.99 (2.73, 3.28)	2.74 (2.33, 3.22)	< 0.001
T2DM	2.86 (2.45, 3.344)	1.43 (1.15, 1.83)	0.003
CVD	1.91 (1.70, 2.13)	0.75 (0.59, 0.96)	0.025
Hypertension	1.76 (1.56, 1.97)	0.69 (0.53, 0.88)	0.004
MetS	5.84 (5.30, 6.45)	4.90 (4.12, 5.82)	< 0.001
**Anthropometric indices**			
BMI (kg/m^2^)	1.78 (1.73, 1.82)	1.86 (1.77, 1.95)	< 0.001
VFA (cm^2^)	1.03 (1.03, 1.03)	1.01 (1.01, 1.02)	< 0.001
**Dietary intake**	1		
Energy intake (Kcal/day)	1.01 (1.01,1.02)	1.01 (0.99, 1.02)	0.884

*Abbreviation* *CVD* cardiovascular disease; *T2DM* type 2 diabetes mellitus; *BMI* body mass index; *WHR* waist hip ratio; *VFA* visceral fat area

*Adjusted for all variables in the table

### Association between the obesity phenotypes and the fatty liver index

Using univariate analysis, compared to the metabolically healthy-non-obese phenotype, the risk of NAFLD increased by 81.32 times in men with the metabolically healthy-obese phenotype, by 6.48 times in those with the metabolically unhealthy-non-obese phenotype, and by 236.70 times in those with the metabolically unhealthy-obese phenotype. Similar results were observed in women **(**Table 4**).**

**Table 4:** Association between the obesity phenotypes and the fatty liver index by logistic regression models

Obesity phenotypes	Model І		Model II	
	OR (95% CI)		OR (95% CI)	
	Men	Women	Men	Women
Metabolically healthy- no obese	1	1	1	1
Metabolically healthy- obese	81.32 (49.51, 133.56)	32.37 (25.93, 40.40)	8.92 (2.20, 15.30)	5.03 (3.86, 6.56)
Metabolically unhealthy- no obese	6.48 (5.43, 7.73)	7.35 (5.97, 9.05)	7.23 (5.82, 8.99)	8.10 (6.34, 10.28)
Metabolically unhealthy- obese	236.70 (115.94, 483.18)	136.62 (101.45, 183.99)	32.97 (15.70, 69.22)	28.60 (20.49, 39.91)

**Model I**: Unadjusted; **Model II**: Adjusted for age, physical activity, SES, smoking, CVD, and VFA

After controlling for confounding variables (Model II), the risk of NAFLD remained significantly increased in men with the metabolically healthy-obese phenotype (8.92 times), the metabolically unhealthy-non-obese phenotype (7.23 times), and the metabolically unhealthy-obese phenotype (32.97 times) compared to the metabolically healthy-non-obese phenotype. Similar results were observed in women **(**Table 4**).**

Generally, the presence of either obesity or an unhealthy metabolism alone, or the simultaneous existence of both these conditions, significantly increases the odds of developing a NAFLD in both women and men.

## Discussion

The study findings support the notion that both obesity and metabolic disorders independently contribute to an increased risk of NAFL. Moreover, the risk of NAFLD was significantly higher in individuals with the metabolically healthy/obese, metabolically unhealthy/non-obese, and metabolically unhealthy/obese phenotypes compared to those with the non-obese/metabolically healthy phenotype. These findings suggest a dose-response relationship between MetS and obesity in the prevalence of NAFLD.

The scientific literature consistently demonstrates a strong association between obesity and NAFLD [13, 14]. Numerous studies have also investigated the relationship between metabolic diseases and fatty liver. NAFLD is more prevalent among individuals who are obese and those with type 2 diabetes, regardless of obesity status [27]. The common metabolic risk factors shared between NAFLD and type 2 diabetes likely contribute to this association [28, 29]. Additionally, dyslipidemia has been identified as a common risk factor for NAFLD and plays a role in increasing the risk of cardiovascular disease in individuals with NAFLD [30, 31]. Hypertension has also been implicated as a potential independent risk factor for NAFLD, with blood pressure control potentially aiding in the prevention or management of NAFLD in non-obese hypertensive patients [32]. A meta-analysis study by Li et al. (2022) has shown a bidirectional relationship between hypertension and NAFLD [33]. However, some studies have not found a significant link between high blood pressure and NAFLD. [34]. In summary, accumulated evidence from previous studies consistently highlights obesity and metabolic disorders as major risk factors for NAFLD. These findings emphasize the importance of addressing these risk factors in the prevention and management of NAFLD.

The combination of obesity and metabolic disorders has a synergistic and dose-response effect on the development of NAFLD. In men, the risk of NAFLD was found to increase by 8.92 times for those with the metabolically healthy/obese phenotype, by 7.23 times for those with the metabolically unhealthy/non-obese phenotype, and by 32.97 times for those with the metabolically unhealthy/obese phenotype compared to individuals with the metabolically healthy/non-obese phenotype. A study by Kuang et al. (2022) demonstrated that both the metabolically healthy/abdominal obesity and metabolically unhealthy/abdominal obesity phenotypes were closely associated with an increased risk of NAFLD in both sexes [35]. Lee et al.‘s (2021) study conducted in Taiwan found a positive association between MetS and obesity with advanced fibrosis. Regardless of metabolic status, obese individuals had a higher percentage of moderate to severe NAFLD. The authors concluded that obesity alone has more deleterious effects than metabolic disorders on the severity of advanced fibrosis, and these effects were greater in women than in men [36]. These findings are consistent with the results of this study. Furthermore, a cohort study of 31,010 adults showed that the metabolically healthy/obese phenotype was associated with higher risks of NAFLD and fibrosis progression, which is in line with the findings of this study. Overall, these results highlight the importance of addressing both obesity and metabolic disorders in the prevention and management of NAFLD and its adverse consequences [37].

The most probable mechanism for the joint effect of obesity and metabolic disorders on fatty liver is inflammation. Obesity and increased visceral fat lead to metabolic abnormalities and insulin resistance [38], which can cause inflammatory processes and accelerate the pathogenesis and progression of NAFLD by increasing free fatty acids [39]. The transfer of free fatty acids from adipose tissues into the liver can cause lipid peroxidation, increase pro-inflammatory cytokines, and ultimately cause liver damage [40]. NAFLD and metabolic disorders share common risk factors, and liver damage can be the result of mechanisms caused by obesity, including oxidative stress, lipotoxicity, pro-inflammatory state, and activation of the renin-angiotensin axis, all of which play an important role in causing metabolic disorders [41]. Therefore, obesity and metabolic disorders lead to fatty liver by creating inflammatory conditions for liver tissues.

The findings of this study showed that the intake of daily energy, hydrogenated fats, total saturated fatty acids, cholesterol, and butter was lower among the obese/metabolically unhealthy group compared to the non-obese/metabolically healthy group. This finding can be explained by the fact that the obese/unhealthy subjects were aware of their metabolic condition. As a result, these individuals were using medication to control their metabolic disorders, such as blood sugar, blood pressure, and lipid levels. They had also modified their dietary patterns as per their doctor’s advice, leading to a reduction in their daily energy intake. In contrast, the healthy/non-obese group maintained their usual eating routine. This difference in the dietary changes between the two groups could be the most important reason for the observed findings in the study.

Obesity and metabolic abnormalities are strongly influenced by lifestyle factors. The study findings showed that physical activity levels were significantly lower in participants with NAFLD, and the prevalence of NAFLD was higher in urban areas compared to rural areas. Additionally, participants with NAFLD had higher energy intake and higher SES compared to those without NAFLD. A study by Darbandi et al. (2021) demonstrated that a pro-inflammatory diet significantly increases the risk of NAFLD [42]. On the other hand, high physical activity has been shown to reduce the risk of NAFLD according to the results of Kemari et al.‘s (2023) study [43]. The prevalence of NAFLD is reported to be higher in urban residents than in rural areas [43], and good SES and high energy intake have been associated with an increased risk of NAFLD [43, 44]. Therefore, lifestyle modifications such as weight control and prevention of metabolic disorders can help prevent and control NAFLD.

This study had several limitations that should be considered. First, inflammatory markers and insulin levels were not measured, which could have provided additional insights into the underlying mechanisms of the joint effect of obesity and metabolic disorders on fatty liver. Second, the diagnosis of fatty liver was based on a non-invasive method rather than using more definitive techniques such as ultrasound or biopsy. While this non-invasive method is acceptable, applicable, and ethical for large population studies, it may have limitations in accurately identifying all cases of fatty liver. It is important to note that this study was cross-sectional in nature, which means that the observed associations between obesity, metabolic disorders, and NAFLD are not causal. Further longitudinal studies are needed to establish a causal relationship. However, the large sample size of the study allowed for control of potential confounding factors, enhancing the robustness of the findings.

## Conclusion

The findings of this study demonstrated that the incidence of NAFLD is significantly higher in individuals with the metabolically healthy/obese, metabolically unhealthy/non-obese, and metabolically unhealthy/obese phenotypes compared to those with the non-obese/metabolically healthy phenotype. Given the high prevalence of these two conditions in the Iranian population, these findings underscore the importance of health promotion strategies aimed at mitigating the adverse consequences of MetS and obesity.

Early screening and diagnosis of NAFLD is crucial to prevent its serious complications like cirrhosis and liver cancer. Therefore, it is essential to raise awareness among the general public and healthcare providers about the importance of lifestyle modifications such as weight management, physical activity, and healthy eating habits in preventing and managing NAFLD. Additionally, policy-level interventions to improve access to healthy food options and promote physical activity can also play a vital role in addressing NAFLD.

## Data Availability

The data analyzed in the study are available from the corresponding author upon reasonable request.

## Abbreviations

RaNCD: Ravansar Non Communicable Diseases
NAFLD: Non alcoholic fatty liver disease
FLI: Fatty liver index
HIS: Hepatic steatosis index
ST: Steato Test
NASH: Non-alcoholic steatohepatitis
T2DM: Type 2 diabetes mellitus
CVDs: Cardiovascular diseases
MetS: Metabolic syndrome
PERSIAN: Prospective Epidemiological Research Studies
SES: Socio-economic status
PCA: Principal component analysis
BMI: Body mass index
BFM: Body fat mass
VFA: Visceral fat area
WHR: Waist-to-hip ratio
WC: Waist circumference
IDF: International Diabetes Federation
ALT: Alanine aminotransferase
AST: Aspartate aminotransferase
GGT: Gamma-glutamyl transferase
